# Supplementary material for: Lipid Variability and Risk of Cardiovascular Diseases and All-Cause Mortality: A Systematic Review and Meta-Analysis of Cohort Studies
Source: Nutrients. 2022 Jun 13;14(12):2450. doi: 10.3390/nu14122450 (PMC9231112; doi:10.3390/nu14122450)
Supplement: Supplementary file 1 [file nutrients-14-02450-s001.zip › nutrients-1731374-supplementary.pdf]

Supplementary Materials

Li et al. Lipid variability and risk of cardiovascular diseases and all-cause mortality: a systematic review and meta-analysis of cohort studies

Table S1. Search strategy to identify studies reporting the associations of lipid variability with cardiovascular disease and mortality

Table S2. Summary of main metrics of LV

Table S3. Quality assessment for cohort studies conducted with the Newcastle Ottawa Scale (NOS)

Table S4. Summary effects and 95% *CI* using random-effects subgroup meta-analysis for the associations of TC variability (top vs. bottom quartiles) with CVDs

Table S5. Summary effects and 95% *CI* using random-effects subgroup meta-analysis for the associations of HDL-C variability (top vs. bottom quartiles) with CVDs

Table S6. Summary effects and 95% *CI* using random-effects subgroup meta-analysis for the associations of LDL-C variability (top vs. bottom quartiles) with CVDs

Table S7. Summary effects and 95% *CI* using random-effects subgroup meta-analysis for the associations of TG variability (top vs. bottom quartiles) with CVDs

Table S8. Summary effects and 95% *CI* using random-effects subgroup meta-analysis for the associations of TC variability (top vs. bottom quartiles) with all-cause mortality

Figure S1. Forest plots of standardized RRs for different types and metrics of TC variability (top vs. bottom quartile) with CVDs

Figure S2. Forest plots of standardized RRs for different types and metrics of HDL-C variability (top vs. bottom quartile) with CVDs

Figure S3. Forest plots of standardized RRs for different types and metrics of LDL-C variability (top vs. bottom quartile) with CVDs

Figure S4. Forest plots of standardized RRs for different types and metrics of TG variability (top vs. bottom quartile) with CVDs

Figure S5. Sensitivity analysis of standardized RRs for different types and metrics of LV (top vs. bottom quartile) and CVDs with excluding the articles counted on patients with hypertension or diabetes

Figure S6. Forest plots of standardized RRs for different types and metrics of TC variability (top vs. bottom quartile) and all-cause mortality

Figure S7. Forest plots of standardized RRs for different types and metrics of HDL-C variability (top vs. bottom quartile) and all-cause mortality

Figure S8. Sensitivity analysis of standardized RRs for different types and metrics of LV (top vs. bottom quartile) and all-cause mortality with excluding the articles counted on patients with hypertension or diabetes

Figure S9. Funnel plots of standardized RRs for different types and metrics of TC variability (top vs. bottom quartile) and CVDs

Figure S10. Funnel plots of standardized RRs for different types and metrics of HDL-C variability (top vs. bottom quartile) and CVDs

Figure S11. Funnel plots of standardized RRs for different types and metrics of LDL-C variability (top vs. bottom quartile) and CVDs

Figure S12. Funnel plots of standardized RRs for different types and metrics of TG variability (top vs. bottom quartile) and CVDs

Figure S13. Funnel plots of standardized RRs for different types and metrics of TC variability (top vs. bottom quartile) and all-cause mortality

Table S1. Search strategy to identify studies reporting the associations of lipid variability with cardiovascular disease and mortality

Database	Search terms	
**PubMed**	#1	search (Lipids[Mesh] OR Cholesterol[Mesh] OR Total Cholesterol[Title/Abstract] OR TC[Title/Abstract] OR Cholesterol, HDL[Mesh] OR High-density Lipoprotein Cholesterol[Title/Abstract] OR HDL-C[Title/Abstract] OR HDL-Cholesterol[Title/Abstract] OR Cholesterol, LDL[Mesh] OR Low-density Lipoprotein Cholesterol[Title/Abstract] OR LDL-C[Title/Abstract] OR LDL-Cholesterol[Title/Abstract] OR Triglycerides[Mesh] OR TG[Title/Abstract])
	#2	search (Variabilit*[Title/Abstract] OR Variation*[Title/Abstract])
	#3	search (#1 AND #2)
	#4	search (Cohort Studies[Mesh] OR Longitudinal Studies[Mesh] OR Follow-Up Studies[Mesh] OR Prospective Studies[Mesh] OR Cohort*[Title/Abstract] OR longitudinal*[Title/Abstract] OR follow-up*[Title/Abstract] OR prospective[Title/Abstract] OR inciden*[Title/Abstract])
	#5	search ((Cardiovascular diseases[Mesh] OR cardiovascular disease*[Title/Abstract] OR CVD[Title/Abstract] OR cardiocerebrovascular disease*[Title/Abstract] OR cardiovascular event*[Title/Abstract] OR heart disease*[Title/Abstract]) OR Stroke[Mesh] OR (Myocardial infarction[Mesh] OR MI[Title/Abstract] OR heart infarction[Title/Abstract]) OR (Myocardial infarction[Mesh] OR ischaemic heart disease*[Title/Abstract] OR ischemic heart disease*[Title/Abstract] OR IHD[Title/Abstract]) OR (Heart Failure[Mesh] OR cardiac failure[Title/Abstract]) OR (Coronary Artery Disease[Mesh] OR coronary artery disease*[Title/Abstract] OR CAD[Title/Abstract] OR coronary heart disease*[Title/Abstract] OR CHD[Title/Abstract]) OR (Cerebral Arterial Diseases[Mesh] OR Cerebrovascular Disorders[Mesh] OR Cerebrovascular Disorder*[Title/Abstract] OR cerebrovascular disease*[Title/Abstract] OR CBVD[Title/Abstract]) OR (Cerebral infarction[Mesh] OR cerebrovascular accident*[Title/Abstract] OR cerebrovascular attack*[Title/Abstract] OR CVA[Title/Abstract] OR cerebral arterial disease*[Title/Abstract]) OR (Peripheral Vascular Diseases[Mesh] OR Peripheral Arterial Disease[Mesh] OR peripheral vascular disease*[Title/Abstract] or peripheral arterial disease*[Title/Abstract] or PVD[Title/Abstract] or PAD[Title/Abstract]))
	#6	search (Mortality[Mesh] OR Death[Mesh] OR Mortalit*[Title/Abstract] OR death*[Title/Abstract] OR fetal[Title/Abstract] OR fatalit*[Title/Abstract])
	#7	search (#5 OR #6)
	#8	search (#3 AND #4 AND #7)
**Medline**	#1	exp Lipids/
	#2	exp Cholesterol/
	#3	(Cholesterol or Total Cholesterol or TC).ab,ti.
	#4	2 or 3

Database	Search terms	
	#5	exp Cholesterol, HDL/
	#6	(High-density Lipoprotein Cholesterol or HDL-C or HDL-Cholesterol).ab,ti.
	#7	5 or 6
	#8	exp Cholesterol, LDL/
	#9	(Low-density Lipoprotein Cholesterol or LDL-C or LDL-Cholesterol).ab,ti.
	#10	8 or 9
	#11	exp Triglycerides/
	#12	(Triglyceride* OR TG).ab,ti.
	#13	11 or 12
	#14	(variability or variation*).ab,ti.
	#15	exp Cohort Studies/
	#16	exp Longitudinal Studies/
	#17	exp Follow-Up Studies/
	#18	exp Prospective Studies/
	#19	(Cohort* or longitudinal* or follow-up* or prospective or inciden*).ab,ti.
	#20	1 or 4 or 7 or 10 or 13
	#21	15 or 16 or 17 or 18 or 19
	#22	exp cardiovascular diseases/
	#23	exp heart diseases/
	#24	(cardiovascular disease* or CVD or cardiocerebrovascular disease* or cardiovascular event* or heart disease*).ab,ti.
	#25	22 or 23 or 24
	#26	exp Stroke/
	#27	exp myocardial infarction/
	#28	(myocardial infarction or MI or heart infarction).ab,ti.
	#29	27 or 28
	#30	exp Myocardial Ischemia/
	#31	(ischaemic heart disease* or ischemic heart disease* or IHD or myocardial ischaemia or myocardial ischemia).ab,ti.
	#32	30 or 31
	#33	exp Heart Failure/
	#34	(heart failure or cardiac failure).ab,ti.
	#35	33 or 34
	#36	exp Coronary Artery Disease/
	#37	(coronary artery disease* or CAD or coronary heart disease* or CHD).ab,ti.
	#38	36 or 37
	#39	exp Cerebrovascular Disorders/
	#40	exp Cerebral Arterial Diseases/

Database	Search terms	
	#41	(Cerebrovascular Disorder* OR cerebrovascular disease* OR CBVD OR Cerebral arterial disease*).ab,ti.
	#42	39 or 40 or 41
	#43	(Cerebrovascular accident* OR cerebrovascular attack* OR CVA).ab,ti.
	#44	exp Cerebral Infarction/
	#45	exp Peripheral Vascular Diseases/
	#46	exp Peripheral Arterial Disease/
	#47	(peripheral vascular disease* or peripheral arterial disease* or PVD or PAD).ab,ti.
	#48	45 or 46 or 47
	#49	25 or 26 or 29 or 32 or 35 or 38 or 42 or 43 or 44 or 48
	#50	exp Mortality/
	#51	exp Death/
	#52	(Mortalit* OR death* OR fetal OR fatalit*).ab,ti.
	#53	50 or 51 or 52
	#54	49 or 53
	#55	14 and 20
	#56	21 and 54 and 55
	#57	limit 56 to (english language and medline)
**EMBASE**	#1	'Lipid blood level'/exp
	#2	'Lipid*':ab,ti OR 'Lipid blood level':ab,ti
	#3	#1 OR #2
	#4	'Cholesterol blood level'/exp
	#5	'Total Cholesterol':ab,ti OR ' TC':ab,ti
	#6	#4 OR #5
	#7	'High density lipoprotein cholesterol'/exp
	#8	'High-density Lipoprotein Cholesterol':ab,ti OR 'HDL-C':ab,ti OR 'HDL-Cholesterol':ab,ti
	#9	#7 OR #8
	#10	'Low density lipoprotein cholesterol'/exp
	#11	'Low-density Lipoprotein Cholesterol':ab,ti OR 'LDL-C':ab,ti OR 'LDL-Cholesterol':ab,ti
	#12	#10 OR #11
	#13	'Triacylglycerol blood level'/exp
	#14	'Triglyceride*':ab,ti OR 'TG':ab,ti
	#15	#13 OR #14
	#16	'variabilit*':ab,ti OR 'variation*':ab,ti
	#17	'Longitudinal Study'/exp
	#18	'Prospective Study'/exp
	#19	'Cohort*':ab,ti OR 'longitudinal*':ab,ti OR 'follow-up*':ab,ti OR

Database	Search terms	
		'prospective':ab,ti OR 'inciden*':ab,ti
	#20	#3 OR #6 OR #9 OR #12 OR #15
	#21	#17 OR #18 OR #19
	#22	#16 AND #20
	#23	'cardiovascular disease'/exp
	#24	'heart disease'/exp
	#25	'cardiovascular disease*':ab,ti OR 'CVD':ab,ti OR 'cardiocerebrovascular disease*':ab,ti OR 'cardiovascular event*':ab,ti OR 'heart disease*':ab,ti
	#26	#23 OR #24 OR #25
	#27	'stroke*':ab,ti
	#28	'heart infarction'/exp
	#29	'myocardial infarction':ab,ti OR 'MI':ab,ti OR 'heart infarction*':ab,ti
	#30	#28 OR #29
	#31	'ischemic heart disease'/exp
	#32	'ischaemic heart disease*':ab,ti OR 'ischemic heart disease*':ab,ti OR 'IHD':ab,ti OR 'myocardial ischaemia':ab,ti OR 'myocardial ischemia':ab,ti
	#33	#31 OR #32
	#34	'heart failure'/exp
	#35	'heart failure':ab,ti OR 'cardiac failure':ab,ti
	#36	#34 OR #35
	#37	'coronary artery disease'/exp
	#38	'coronary artery disease*':ab,ti OR 'CAD':ab,ti OR 'coronary heart disease*':ab,ti OR 'CHD':ab,ti
	#39	#37 OR #38
	#40	'cerebrovascular disease'/exp
	#41	'Cerebrovascular Disorder*':ab,ti OR 'cerebrovascular disease*':ab,ti OR 'CBVD':ab,ti OR 'cerebral arterial disease*':ab,ti
	#42	#40 OR #41
	#43	'cerebrovascular accident'/exp
	#44	'Cerebrovascular accident*':ab,ti OR 'cerebrovascular attack*':ab,ti OR 'CVA':ab,ti
	#45	#43 OR #44
	#46	'cerebral infarction':ab,ti
	#47	'peripheral vascular disease'/exp
	#48	'peripheral vascular disease*':ab,ti OR 'peripheral arterial disease*':ab,ti OR 'PVD':ab,ti OR 'PAD':ab,ti
	#49	#46 OR #47 OR #48
	#50	'mortality'/exp
	#51	'death'/exp
	#52	'fatality'/exp
	#53	'Mortalit*':ab,ti OR 'death*':ab,ti OR 'fetal':ab,ti OR 'fatalit*':ab,ti

Database	Search terms	
	#54	#50 OR #51 OR #52 OR #53
	#55	#26 OR #27 OR #30 OR #33 OR #36 OR #39 OR #42 OR #45 OR #46 OR #49
	#56	#54 OR #55
	#57	#21 AND #22 AND #56
	#58	#57 AND [embase]/lim AND ([article]/lim OR [article in press]/lim OR [short survey]/lim) AND [english]/lim

Note: the above databases were searched in 14 Dec 2021 before manuscript submission.

Table S2. Summary of main metrics of LV

LV metrics	Definition	Calculation
**TC**		
TC-CV	Coefficient of variation of TC	CV= $\frac{SD}{mean}$
TC-SD	Standard deviation of TC	SD= $\sqrt{\frac{\sum\lvert x-\mu\rvert^2}{n}}$ (x: value in the data set; μ: mean; n: the number of measurements)
TC-VIM	Variation independent of mean of TC	VIM= $\frac{SD}{mean^{\beta}}$ (β: regression coefficient based on the natural logarithm of SD over the natural logarithm of the mean)
TC-ARV	Average real variability of TC	ARV= $\frac{1}{n-1}\Sigma_{i=1}^{n-1}\lvert Value_{i+1} - Value_{i}\rvert$ (n: number of measurements)
TC-ASV	Average successive variability of TC	ASV= $\frac{1}{n}\sum\lvert Value_{i} - Value_{i+1}\rvert$ (n: number of measurements)
TC-RMSE	Root Mean Square Error of TC	RMSE= $\sqrt{\frac{1}{n}\sum_{i=1}^{n}(\widehat{y_i} - y_i)^2}$ ($\widehat{y_i}$: predicted value; y_i: true value)
TC-SDR	Standard deviation of the residuals from the linear regression of the lipid measures of TC	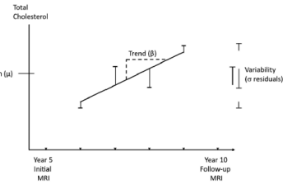 (μ: mean; β: intraindividual trend over time, or slope; σ: standard deviation)
**HDL-C**		
HDL-C-CV	Coefficient of variation of HDL-C	CV= $\frac{SD}{mean}$
HDL-C-SD	Standard deviation of HDL-C	SD= $\sqrt{\frac{\sum\lvert x-\mu\rvert^2}{n}}$ (x: value in the data set; μ: mean; n: the number of measurements)
HDL-C-VIM	Variation independent of mean of HDL-C	VIM= $\frac{SD}{mean^{\beta}}$ (β: regression coefficient based on the natural logarithm of SD over the natural logarithm of the mean)
HDL-C-ARV	Average real variability of HDL-C	ARV= $\frac{1}{n-1}\Sigma_{i=1}^{n-1}\lvert Value_{i+1} - Value_{i}\rvert$ (n: number of measurements)
**LDL-C**		
LDL-C-CV	Coefficient of variation of LDL-C	CV= $\frac{SD}{mean}$

LDL-C-SD	Standard deviation of LDL-C	SD=$\sqrt{\frac{\Sigma\lvert x-\mu\rvert^2}{n}}$ (x: value in the data set; μ: mean; n: the number of measurements)
LDL-C-VIM	Variation independent of mean of LDL-C	VIM=$\frac{SD}{mean^{\beta}}$ (β: regression coefficient based on the natural logarithm of SD over the natural logarithm of the mean)
LDL-C-ARV	Average real variability of LDL-C	ARV=$\frac{1}{n-1}\Sigma_{i=1}^{n-1}\lvert Value_{i+1}-Value_i\rvert$ (n: number of measurements)
**TG**		
TG-CV	Coefficient of variation of TG	CV=$\frac{SD}{mean}$
TG-SD	Standard deviation of TG	SD=$\sqrt{\frac{\Sigma\lvert x-\mu\rvert^2}{n}}$ (x: value in the data set; μ: mean; n: the number of measurements)
TG-VIM	Variation independent of mean of TG	VIM=$\frac{SD}{mean^{\beta}}$ (β: regression coefficient based on the natural logarithm of SD over the natural logarithm of the mean)
TG-ARV	Average real variability of TG	ARV=$\frac{1}{n-1}\Sigma_{i=1}^{n-1}\lvert Value_{i+1}-Value_i\rvert$ (n: number of measurements)

Notes: LV, lipid variability; TC, total cholesterol; HDL-C, high-density lipoprotein cholesterol; LDL-C, low-density lipoprotein cholesterol; TG, triglycerides.

Table S3. Quality assessment for cohort studies conducted with the Newcastle Ottawa Scale (NOS)

Publications	Selection ☆☆☆☆/★★★★				Comparability ☆☆/★★	Outcome ☆☆☆/★★★			Overall stars	Quality assessment
	Representativeness of the exposed cohort	Selection of the non-exposed cohort	Ascertainment of exposure	Demonstration that outcome of interest was not present at the start of the study	Comparability of cohorts on the basis of the design or analysis controlled for confounders	Assessment of outcome	Was follow-up long enough for outcomes to occur	Adequacy of follow-up of cohorts		
Kreger et al (1994)[1]	★	★	★	★	☆☆	★	★	★	7/9	Good quality
Kim et al (2017)[2]	★	★	★	★	★★	★	★	☆	8/9	Good quality
Kwon et al (2019)[3]	★	★	★	★	★☆	★	★	☆	7/9	Good quality
Zhu et al (2019)[4]	★	★	★	★	★☆	★	☆	☆	6/9	Fair quality
Lee et al (2019)[5]	★	★	★	★	★☆	★	★	☆	7/9	Good quality
Liu et al (2020)[6]	★	★	★	★	★★	★	★	★	9/9	Good quality
Han et al (2020)[7]	★	★	★	★	★☆	★	★	☆	7/9	Good quality
Kalani et al (2020)[8]	☆	★	★	★	★☆	★	★	★	7/9	Good quality
Wang et al (2020)[9]	★	★	★	★	★☆	★	★	★	8/9	Good quality
Wan, et al. (2020)[10]	☆	★	★	★	★★	★	★	☆	7/9	Good quality
Huang, et al. (2021)[11]	☆	★	★	★	★☆	★	☆	☆	5/9	Fair quality

Note: (a)good quality if NOS≥7 stars, (b) fair quality if NOS 4-6 stars, and (c) poor quality if NOS≤4 stars.

Table S4. Summary effects and 95% CI using random-effects subgroup meta-analysis for the associations of TC variability (top vs. bottom quartile) with CVDs

Characteristics of studies and populations	Number of Data Points	SRR (95% *CI*)	I^2 (%)	*p* value: *Q* test	*p* value: Subgroup difference	Number of Data Points	SRR (95% *CI*)	I^2 (%)	*p* value: *Q* test	*p* value: Subgroup difference	Number of Data Points	SRR (95% *CI*)	I^2 (%)	*p* value: *Q* test	*p* value: Subgroup difference
	**TC-CV**					**TC-SD**					**TC-VIM**				
Global analysis	7	1.29 (1.15,1.45)				7	1.28 (1.15,1.43)				7	1.26 (1.13,1.41)			
Subtypes of CVDs					0.974					0.822					0.969
MI	2	1.39 (1.03,1.87)	76.3	0.040		2	1.35 (1.03,1.77)	79.7	0.007		2	1.39 (1.08,1.79)	67.3	0.080	
Stroke	3	1.56 (1.07,2.28)	90.7	0.001		3	1.59 (1.12,2.27)	88.8	<0.001		3	1.49 (1.06,2.10)	88.3	<0.001	
AF	1	1.10 (1.06,1.13)	/	/		1	1.09 (1.06,1.13)	/	/		1	1.08 (1.04,1.12)	/	/	
HF	1	1.17 (1.13,1.22)	/	/		1	1.18 (1.13,1.23)	/	/		1	1.17 (1.12,1.22)	/	/	
Gender[a]					0.187					0.093					0.035
Male	4	1.08 (1.05,1.11)	54.2	0.088		3	1.09 (1.07,1.10)	0.0	0.557		3	1.08 (1.07,1.10)	0.0	0.513	
Female	4	1.09 (0.99,1.19)	89.5	<0.001		3	1.06 (1.03,1.08)	0.0	0.942		3	1.05 (1.01,1.09)	0.0	0.924	

Characteristics of studies and populations	Number of Data Points	SRR (95% *CI*)	I^2 (%)	*p* value: *Q* test	*p* value: Subgroup difference	Number of Data Points	SRR (95% *CI*)	I^2 (%)	*p* value: *Q* test	*p* value: Subgroup difference	Number of Data Points	SRR (95% *CI*)	I^2 (%)	*p* value: *Q* test	*p* value: Subgroup difference
	**TC-CV**					**TC-SD**					**TC-VIM**				
Adjusted for mean lipid level					0.003					0.001					0.002
Yes	6	1.25 (1.12,1.40)	92.3	<0.001		6	1.24 (1.13,1.37)	89.8	<0.001		6	1.23 (1.11,1.36)	90.7	<0.001	
No	1	3.83 (2.03,7.25)	/	/		1	4.43 (2.29,8.56)	/	/		1	3.87 (2.04,7.32)	/	/	
Adjusted for lipid-lowering medication					0.216					<0.001					<0.001
Yes	5	1.43 (1.17,1.75)	85.6	<0.001		4	1.52 (1.23,1.86)	78.9	0.003		4	1.49 (1.23,1.81)	77.9	0.004	
No	2	1.13 (1.06,1.21)	84.5	0.011		3	1.13 (1.07,1.21)	72.7	0.026		3	1.12 (1.04,1.19)	76.0	0.016	

Note: The variables used for subgroup meta-analysis included: study design, subtypes of CVDs, gender (male or female), whether adjusting for mean lipid level or not, whether adjusting for lipid-lowering medication or not, whether adjusting for hypertension or not, whether adjusting for diabetes or not, whether adjusting for BMI or not, and whether adjusting for smoking or not. All studies of TC-CV, TC-SD and TC-VIM with CVDs were adjusted for hypertension, diabetes, BMI and smoking (data not shown).

SRR, summary relative risk; CI, confidence interval; CVDs, cardiovascular diseases; TC, total cholesterol; CV, coefficient of variation; SD, standard deviation; VIM, variation independent of mean; MI, myocardial infarction; AF, atrial fibrillation; HF, heart failure; [a]Three studies have explored the relationships between TC-CV, TC-SD, TC-VIM and CVDs in males and females.

Table S5. Summary effects and 95% CI using random-effects subgroup meta-analysis for the associations of HDL-C variability (top vs. bottom quartile) with CVDs

Characteristics of studies and populations	HDL-C-CV					HDL-C-SD					HDL-C-VIM				
	Number of Data Points	SRR (95% *CI*)	I^2 (%)	*p* value: *Q* test	*p* value: Subgroup difference	Number of Data Points	SRR (95% *CI*)	I^2 (%)	*p* value: *Q* test	*p* value: Subgroup difference	Number of Data Points	SRR (95% *CI*)	I^2 (%)	*p* value: *Q* test	*p* value: Subgroup difference
Global analysis	6	1.11 (1.07,1.15)				4	1.18 (1.02,1.38)				6	1.18 (1.09,1.27)			
Subtypes of CVDs					0.636					0.613					0.389
MI	2	1.28 (0.94,1.75)	79.1	0.029		1	1.46 (1.08,1.97)	/	0.007		2	1.26 (1.23,1.30)	0.0	0.080	
Stroke	3	1.11 (1.09,1.14)	0.0	0.557		2	1.22 (1.04,1.44)	0.0	<0.001		3	1.15 (1.10,1.19)	4.4	<0.001	
AF	1	1.06 (1.03,1.10)	/	/		1	1.06 (1.02,1.10)	/	/		1	1.07 (1.03,1.11)	/	/	
Gender[a]					0.134					/					/
Male	3	1.08 (1.06,1.09)	38.1	0.199		1	1.04 (1.00,1.08)	/	/		1	1.06 (1.02,1.10)	/	/	
Female	3	1.10 (1.06,1.14)	75.2	0.018		1	1.12 (1.04,1.20)	/	/		1	1.10 (1.02,1.18)	/	/	
Adjusted for mean lipid level					0.020					0.334					0.349

Characteristics of studies and populations	Number of Data Points	SRR (95% *CI*)	I^2 (%)	*p* value: *Q* test	*p* value: Subgroup difference	Number of Data Points	SRR (95% *CI*)	I^2 (%)	*p* value: *Q* test	*p* value: Subgroup difference	Number of Data Points	SRR (95% *CI*)	I^2 (%)	*p* value: *Q* test	*p* value: Subgroup difference
	**HDL-C-CV**					**HDL-C-SD**					**HDL-C-VIM**				
Yes	3	1.19 (1.00,1.42)	75.6	0.017		1	1.42 (0.80,2.52)	/	/		3	1.15 (1.01,1.30)	56.2	0.102	
No	3	1.12 (1.10,1.14)	0.0	0.443		3	1.17 (1.00,1.38)	69.6	0.037		3	1.21 (1.10,1.33)	93.8	<0.001	
Adjusted for lipid-lowering medication					0.004					0.023					0.132
Yes	5	1.13 (1.09,1.17)	43.9	0.129		2	1.45 (1.11,1.89)	0.0	0.935		4	1.22 (1.11,1.34)	90.9	<0.001	
No	1	1.06 (1.03,1.10)	/	/		2	1.10 (0.98,1.24)	57.6	0.125		2	1.11 (0.99,1.23)	51.5	0.151	

Note: The variables used for subgroup meta-analysis included: study design, subtypes of CVDs, gender (male or female), whether adjusting for mean lipid level or not, whether adjusting for lipid-lowering medication or not, whether adjusting for hypertension or not, whether adjusting for diabetes or not, whether adjusting for BMI or not, and whether adjusting for smoking or not. All studies of HDL-C-CV, HDL-C-SD and HDL-C-VIM with CVDs were adjusted for hypertension, diabetes, BMI and smoking (data not shown).

SRR, summary relative risk; CI, confidence interval; CVDs, cardiovascular diseases; HDL-C, high-density lipoprotein cholesterol; CV, coefficient of variation; SD, standard deviation; VIM, variation independent of mean; MI, myocardial infarction; AF, atrial fibrillation; [a]Two studies have explored the relationship between HDL-C-CV and CVDs in males and females; One study have explored the relationship between HDL-C-SD and CVDs in males and females; One study have explored the relationship between HDL-C-VIM and CVDs in males and females.

Table S6. Summary effects and 95% CI using random-effects subgroup meta-analysis for the associations of LDL-C variability (top vs. bottom quartile) with CVDs

**Characteristics of studies and populations**	**Number of Data Points**	**SRR (95% *CI*)**	**I^2 (%)**	***p* value**		**Number of Data Points**	**SRR (95% *CI*)**	**I^2 (%)**	***p* value**		**Number of Data Points**	**SRR (95% *CI*)**	**I^2 (%)**	***p* value**	
				***Q* test**	**Subgroup difference**				***Q* test**	**Subgroup difference**				***Q* test**	**Subgroup difference**
	**LDL-C-CV**					**LDL-C-SD**					**LDL-C-VIM**				
Global analysis	4	1.18 (1.02,1.38)				5	1.09 (1.02,1.17)				4	1.16 (1.02,1.32)			
Subtypes of CVDs					0.890					0.038					0.701
MI	1	1.34 (1.01,1.78)	/	/		1	1.25 (0.93,1.69)	/	/		1	1.22 (0.91,1.63)	/	/	
Stroke	2	1.35 (0.78,2.31)	72.9	0.055		2	1.10 (0.93,1.30)	82.1	0.018		2	1.47 (0.78,2.75)	79.5	0.027	
AF	1	1.06 (1.03,1.10)	/	/		1	1.03 (1.00,1.07)	/	/		1	1.10 (1.06,1.15)	/	/	
Gender[a]					0.873					0.998					0.917
Male	2	1.09 (0.90,1.31)	9.9	0.292		2	1.35 (0.67,2.72)	71.4	0.062		2	1.24 (0.80,1.93)	41.0	0.193	
Female	2	1.54 (0.62,3.83)	81.2	0.021		2	1.26 (0.72,2.22)	64.8	0.092/		2	1.51 (0.69,3.32)	77.5	0.035	
Adjusted for mean lipid level					0.039					0.011					0.019

Characteristics of studies and populations	Number of Data Points	SRR (95% *CI*)	I^2 (%)	*p* value: *Q* test	*p* value: Subgroup difference	Number of Data Points	SRR (95% *CI*)	I^2 (%)	*p* value: *Q* test	*p* value: Subgroup difference	Number of Data Points	SRR (95% *CI*)	I^2 (%)	*p* value: *Q* test	*p* value: Subgroup difference
	**LDL-C-CV**					**LDL-C-SD**					**LDL-C-VIM**				
Yes	3	1.08 (1.00,1.17)	23.0	0.273		4	1.07 (1.02,1.13)	59.9	0.058		3	1.11 (1.07,1.15)	0.0	0.793	
No	1	1.90 (1.10,3.29)	/	/		1	2.14 (1.26,3.62)	/	/		1	2.15 (1.23,3.73)	/	/	
Adjusted for lipid-lowering medication					0.136					0.008					0.094
Yes	3	1.28 (0.98,1.67)	56.9	0.098		3	1.30 (0.97,1.72)	70.5	0.034		2	1.54 (0.89,2.65)	68.1	0.077	
No	1	1.06 (1.02,1.10)	/	/		2	1.04 (1.00,1.07)	0.0	0.471		2	1.10 (1.06,1.15)	0.0	0.870	

Note: The variables used for subgroup meta-analysis included: subtypes of CVDs, gender (male or female), whether adjusting for mean lipid level or not, whether adjusting for lipid-lowering medication or not, whether adjusting for hypertension or not, whether adjusting for diabetes or not, whether adjusting for BMI or not, and whether adjusting for smoking or not. All studies of LDL-C-CV, LDL-C-SD and LDL-C-VIM with CVDs were adjusted for hypertension, diabetes, BMI and smoking (data not shown).

SRR, summary relative risk; CI, confidence interval; CVDs, cardiovascular diseases; LDL-C, low-density lipoprotein cholesterol; CV, coefficient of variation; SD, standard deviation; VIM, variation independent of mean; MI, myocardial infarction; AF, atrial fibrillation; [a]Two studies have explored the relationship between LDL-C-CV, LDL-C-SD, LDL-C-VIM and CVDs in males and females.

Table S7. Summary effects and 95% CI using random-effects subgroup meta-analysis for the associations of TG variability (top vs. bottom quartile) with CVDs

Characteristics of studies and populations	Number of Data Points	SRR (95% *CI*)	I^2 (%)	*p* value		Number of Data Points	SRR (95% *CI*)	I^2 (%)	*p* value		Number of Data Points	SRR (95% *CI*)	I^2 (%)	*p* value	
				Q test	Subgroup difference				*Q* test	Subgroup difference				*Q* test	Subgroup difference
	TG-CV					TG-SD					TG-VIM				
Global analysis	4	1.04 (0.98,1.11)				5	1.05 (1.02,1.09)				4	1.02 (0.95,1.09)			
Subtypes of CVDs					0.070					0.236					0.433
MI	1	1.21 (0.92,1.59)	/	/		1	1.28 (0.92,1.79)	/	/		1	0.89 (0.66,1.20)	/	/	
Stroke	2	0.92 (0.78,1.09)	0.0	0.849		2	1.08 (0.87,1.34)	6.6	0.301		2	0.92 (0.78,1.08)	0.0	0.693	
AF	1	1.05 (1.02,1.09)	/	/		1	1.04 (1.01,1.08)	/	/		1	1.04 (1.01,1.08)	/	/	
Gender[a]					/					/					/
Male	1	1.07 (1.03,1.11)	/	/		1	1.07 (1.03,1.11)	/	/		1	1.07 (1.02,1.11)	/	/	
Female	1	0.99 (0.92,1.06)	/	/		1	0.97 (0.90,1.04)	/	/		1	0.98 (0.91,1.04)	/	/	
Adjusted for mean lipid level					0.784					0.309					0.410

Characteristics of studies and populations	Number of Data Points	SRR (95% *CI*)	I^2 (%)	*p* value: *Q* test	*p* value: Subgroup difference	Number of Data Points	SRR (95% *CI*)	I^2 (%)	*p* value: *Q* test	*p* value: Subgroup difference	Number of Data Points	SRR (95% *CI*)	I^2 (%)	*p* value: *Q* test	*p* value: Subgroup difference
	**TG-CV**					**TG-SD**					**TG-VIM**				
Yes	3	1.04 (0.94,1.14)	23.0	0.273		4	1.05 (1.02,1.09)	0.0	0.426		3	1.01 (0.93,1.10)	25.9	0.259	
No	1	0.97 (0.57,1.67)	/	/		1	1.42 (0.80,2.52)	/	/		1	0.83 (0.49,1.41)	/	/	
Adjusted for lipid-lowering medication					0.450					0.141					0.198
Yes	3	1.01 (0.84,1.22)	27.4	0.252		3	1.10 (1.03,1.17)	0.0	0.441		2	0.88 (0.68,1.14)	0.0	0.827	
No	1	1.05 (1.02,1.09)	/	/		2	1.04 (1.01,1.08)	0.0	0.905		2	1.02 (0.93,1.12)	40.3	0.196	

Note: The variables used for subgroup meta-analysis included: study design, subtypes of CVDs, gender (male or female), whether adjusting for mean lipid level or not, whether adjusting for lipid-lowering medication or not, whether adjusting for hypertension or not, whether adjusting for diabetes or not, whether adjusting for BMI or not, and whether adjusting for smoking or not. All studies of TG-CV, TG-SD and TG-VIM with CVDs were adjusted for hypertension, diabetes, BMI and smoking (data not shown).

SRR, summary relative risk; CI, confidence interval; CVDs, cardiovascular diseases; TG, triglycerides; CV, coefficient of variation; SD, standard deviation; VIM, variation independent of mean; MI, myocardial infarction; AF, atrial fibrillation; [a]One studies have explored the relationship between TG-CV, TG-SD, TG-VIM and CVDs in males and females.

Table S8. Summary effects and 95% CI using random-effects subgroup meta-analysis for the associations of TC variability (top vs. bottom quartile) with all-cause mortality

Characteristics of studies and populations	TC-CV: Number of Data Points	TC-CV: SRR (95% *CI*)	TC-CV: I^2 (%)	TC-CV: *p* value *Q* test	TC-CV: *p* value Subgroup difference	TC-SD: Number of Data Points	TC-SD: SRR (95% *CI*)	TC-SD: I^2 (%)	TC-SD: *p* value *Q* test	TC-SD: *p* value Subgroup difference	TC-VIM: Number of Data Points	TC-VIM: SRR (95% *CI*)	TC-VIM: I^2 (%)	TC-VIM: *p* value *Q* test	TC-VIM: *p* value Subgroup difference
Global analysis	3	1.28 (1.15,1.42)				3	1.32 (1.22,1.44)				3	1.18 (1.09,1.27)			
Gender[a]					<0.001					/					/
Male	2	1.27 (1.25,1.29)	0.0	0.863		1	1.26 (1.24,1.28)	/	/		1	1.26 (1.24,1.28)	/	/	
Female	2	1.08 (1.05,1.11)	0.0	0.857		1	1.08 (1.05,1.12)	/	/		1	1.08 (1.05,1.11)	/	/	
Adjusted for diabetes					0.603					0.263					0.205
Yes	2	1.30 (1.14,1.49)	76.5	0.039		2	1.36 (1.25,1.48)	42.5	0.187		2	1.35 (1.28,1.41)	0.0	0.357	
No	1	1.21 (1.05,1.40)	/	/		1	1.22 (1.05,1.41)	/	/		1	1.22 (1.06,1.41)	/	/	
Adjusted for lipid-lowering medication					0.603					0.263					0.205

Characteristics of studies and populations	Number of Data Points	SRR (95% *CI*)	I^2 (%)	*p* value: *Q* test	*p* value: Subgroup difference	Number of Data Points	SRR (95% *CI*)	I^2 (%)	*p* value: *Q* test	*p* value: Subgroup difference	Number of Data Points	SRR (95% *CI*)	I^2 (%)	*p* value: *Q* test	*p* value: Subgroup difference
	TC-CV					TC-SD					TC-VIM				
Yes	2	1.30 (1.14,1.49)	76.5	0.039		2	1.36 (1.25,1.48)	42.5	0.187		2	1.35 (1.28,1.41)	0.0	0.357	
No	1	1.21 (1.05,1.40)	/	/		1	1.22 (1.05,1.41)	/	/		1	1.22 (1.06,1.41)	/	/	

Note: The variables used for subgroup meta-analysis included: subtypes of CVDs, gender (male or female), whether adjusting for mean lipid level or not, whether adjusting for lipid-lowering medication or not, whether adjusting for hypertension or not, whether adjusting for diabetes or not, whether adjusting for BMI or not, and whether adjusting for smoking or not. All studies of TC-CV, TC-SD, TC-VIM with all-cause mortality were adjusted for mean lipid level, hypertension, BMI and smoking (data not shown).

SRR, summary relative risk; CI, confidence interval; TC, total cholesterol; TC-CV, coefficient of variation of TC; TC-SD, standard deviation of TC; TC-VIM, variation independent of mean of TC; [a]Two studies have explored the relationships between TC-CV and all-cause mortality in males and females; One study have explored the relationship of TC-SD and TC-VIM with all-cause mortality in males and females

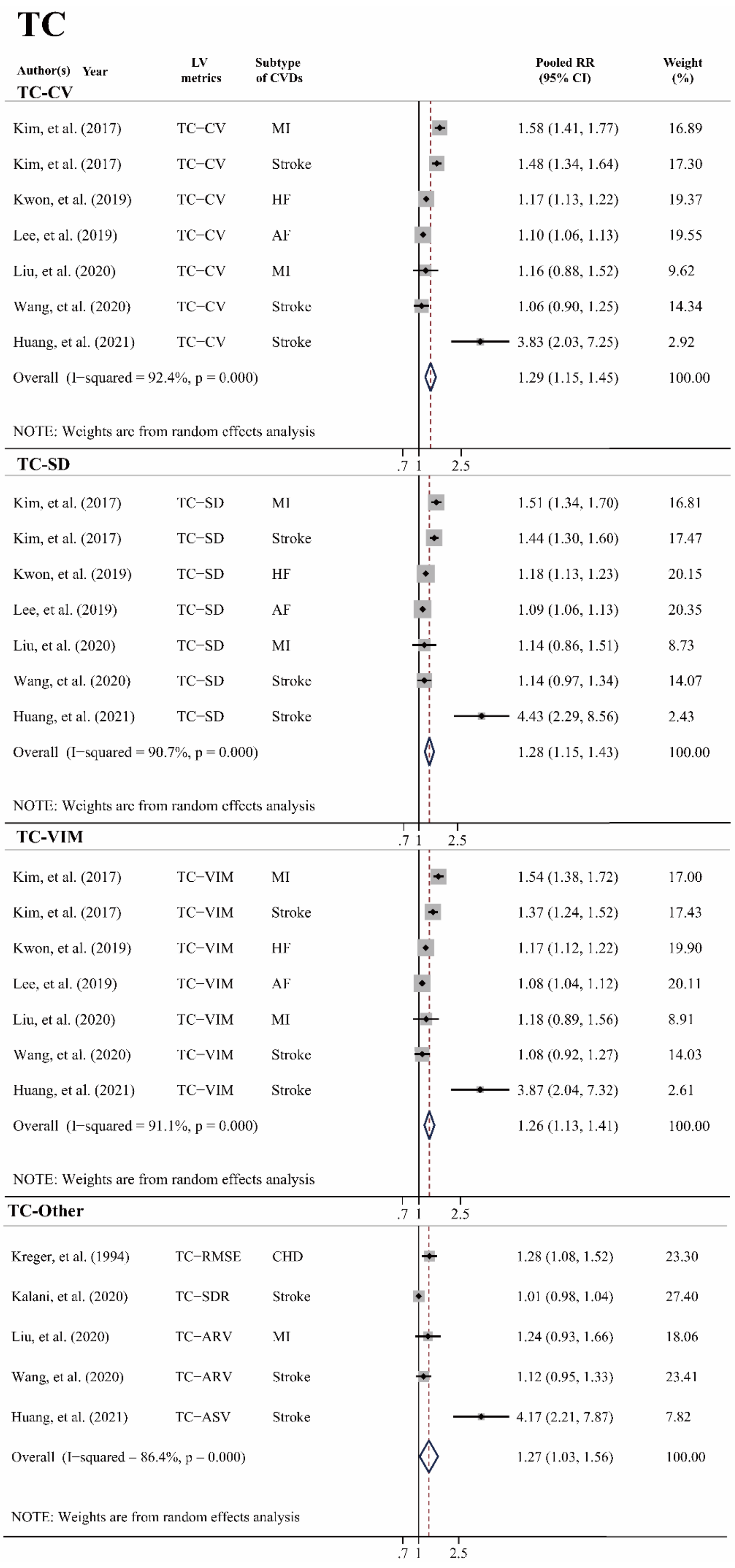
TC
Author(s) Year
LV metrics
Subtype of CVDs
Pooled RR (95% CI)
Weight (%)
TC-CV
Kim, et al. (2017) TC−CV MI 1.58 (1.41, 1.77) 16.89
Kim, et al. (2017) TC−CV Stroke 1.48 (1.34, 1.64) 17.30
Kwon, et al. (2019) TC−CV HF 1.17 (1.13, 1.22) 19.37
Lee, et al. (2019) TC−CV AF 1.10 (1.06, 1.13) 19.55
Liu, et al. (2020) TC−CV MI 1.16 (0.88, 1.52) 9.62
Wang, et al. (2020) TC−CV Stroke 1.06 (0.90, 1.25) 14.34
Huang, et al. (2021) TC−CV Stroke 3.83 (2.03, 7.25) 2.92
Overall (I−squared = 92.4%, p = 0.000) 1.29 (1.15, 1.45) 100.00
NOTE: Weights are from random effects analysis
.7 1 2.5
TC-SD
Kim, et al. (2017) TC−SD MI 1.51 (1.34, 1.70) 16.81
Kim, et al. (2017) TC−SD Stroke 1.44 (1.30, 1.60) 17.47
Kwon, et al. (2019) TC−SD HF 1.18 (1.13, 1.23) 20.15
Lee, et al. (2019) TC−SD AF 1.09 (1.06, 1.13) 20.35
Liu, et al. (2020) TC−SD MI 1.14 (0.86, 1.51) 8.73
Wang, et al. (2020) TC−SD Stroke 1.14 (0.97, 1.34) 14.07
Huang, et al. (2021) TC−SD Stroke 4.43 (2.29, 8.56) 2.43
Overall (I−squared = 90.7%, p = 0.000) 1.28 (1.15, 1.43) 100.00
NOTE: Weights are from random effects analysis
.7 1 2.5
TC-VIM
Kim, et al. (2017) TC−VIM MI 1.54 (1.38, 1.72) 17.00
Kim, et al. (2017) TC−VIM Stroke 1.37 (1.24, 1.52) 17.43
Kwon, et al. (2019) TC−VIM HF 1.17 (1.12, 1.22) 19.90
Lee, et al. (2019) TC−VIM AF 1.08 (1.04, 1.12) 20.11
Liu, et al. (2020) TC−VIM MI 1.18 (0.89, 1.56) 8.91
Wang, et al. (2020) TC−VIM Stroke 1.08 (0.92, 1.27) 14.03
Huang, et al. (2021) TC−VIM Stroke 3.87 (2.04, 7.32) 2.61
Overall (I−squared = 91.1%, p = 0.000) 1.26 (1.13, 1.41) 100.00
NOTE: Weights are from random effects analysis
.7 1 2.5
TC-Other
Kreger, et al. (1994) TC−RMSE CHD 1.28 (1.08, 1.52) 23.30
Kalani, et al. (2020) TC−SDR Stroke 1.01 (0.98, 1.04) 27.40
Liu, et al. (2020) TC−ARV MI 1.24 (0.93, 1.66) 18.06
Wang, et al. (2020) TC−ARV Stroke 1.12 (0.95, 1.33) 23.41
Huang, et al. (2021) TC−ASV Stroke 4.17 (2.21, 7.87) 7.82
Overall (I−squared = 86.4%, p = 0.000) 1.27 (1.03, 1.56) 100.00
NOTE: Weights are from random effects analysis
.7 1 2.5

Figure S1. Forest plots of standardized RRs for different types and metrics of TC variability (top vs. bottom quartile) with CVDs

Notes: CVDs, cardiovascular diseases; RR, relative risk; CI, confidence interval; TC, total cholesterol; TC-CV, coefficient of variation of TC; TC-SD, standard deviation of TC; TC-VIM, variation independent of mean of TC; TC-Other included average real variability of TC (TC-ARV), standard deviation of the residuals of TC (TC-SDR) and root mean square error of TC (TC-RMSE); MI, myocardial infarction; AF, atrial fibrillation; HF, heart failure; CHD, coronary heart disease.

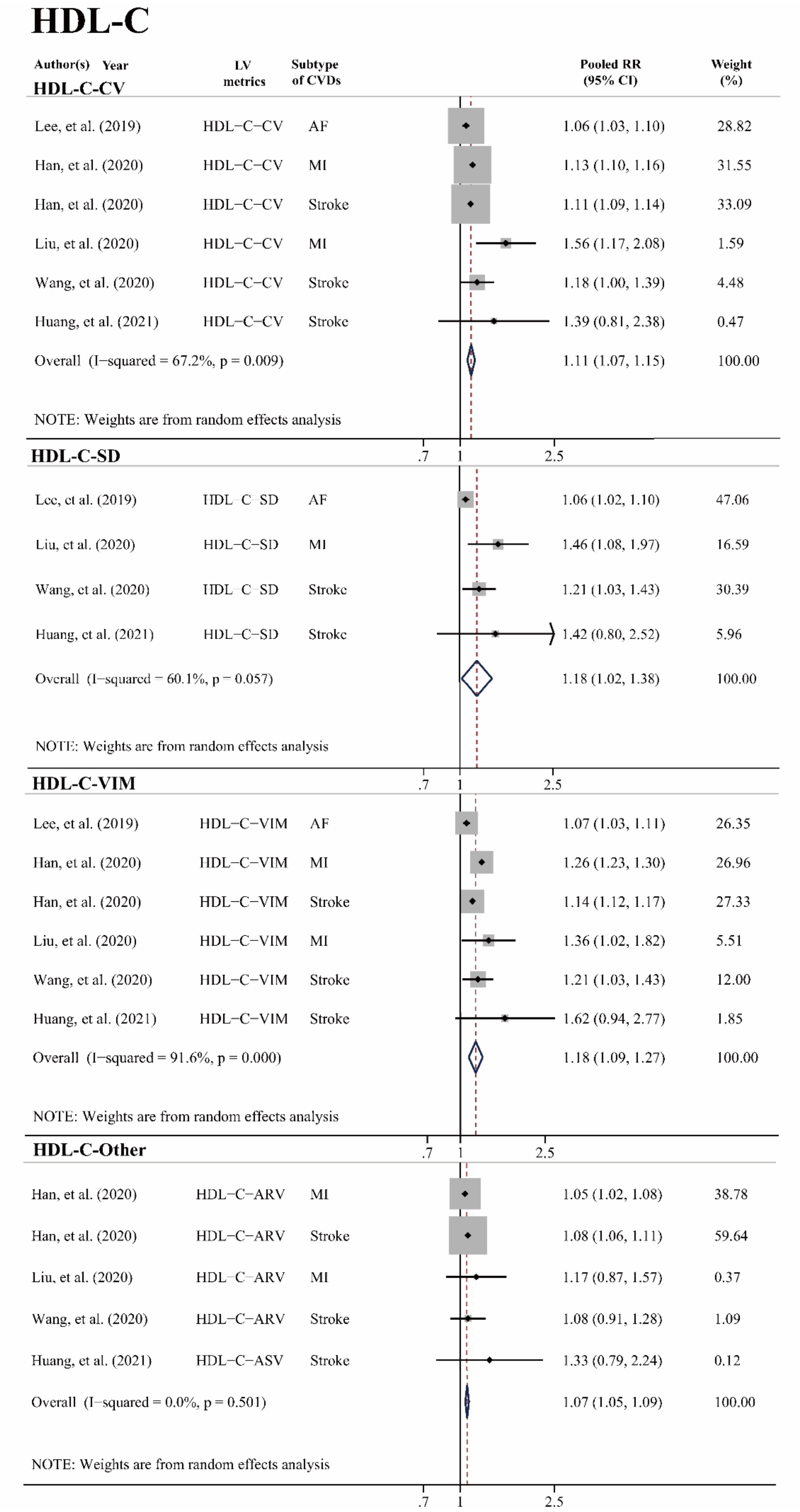
HDL-C
Author(s) Year
LV metrics
Subtype of CVDs
Pooled RR (95% CI)
Weight (%)
HDL-C-CV
Lee, et al. (2019) HDL−C−CV AF 1.06 (1.03, 1.10) 28.82
Han, et al. (2020) HDL−C−CV MI 1.13 (1.10, 1.16) 31.55
Han, et al. (2020) HDL−C−CV Stroke 1.11 (1.09, 1.14) 33.09
Liu, et al. (2020) HDL−C−CV MI 1.56 (1.17, 2.08) 1.59
Wang, et al. (2020) HDL−C−CV Stroke 1.18 (1.00, 1.39) 4.48
Huang, et al. (2021) HDL−C−CV Stroke 1.39 (0.81, 2.38) 0.47
Overall (I−squared = 67.2%, p = 0.009) 1.11 (1.07, 1.15) 100.00
NOTE: Weights are from random effects analysis
.7
1
2.5
HDL-C-SD
Lee, et al. (2019) HDL−C−SD AF 1.06 (1.02, 1.10) 47.06
Liu, et al. (2020) HDL−C−SD MI 1.46 (1.08, 1.97) 16.59
Wang, et al. (2020) HDL−C−SD Stroke 1.21 (1.03, 1.43) 30.39
Huang, et al. (2021) HDL−C−SD Stroke 1.42 (0.80, 2.52) 5.96
Overall (I−squared = 60.1%, p = 0.057) 1.18 (1.02, 1.38) 100.00
NOTE: Weights are from random effects analysis
.7
1
2.5
HDL-C-VIM
Lee, et al. (2019) HDL−C−VIM AF 1.07 (1.03, 1.11) 26.35
Han, et al. (2020) HDL−C−VIM MI 1.26 (1.23, 1.30) 26.96
Han, et al. (2020) HDL−C−VIM Stroke 1.14 (1.12, 1.17) 27.33
Liu, et al. (2020) HDL−C−VIM MI 1.36 (1.02, 1.82) 5.51
Wang, et al. (2020) HDL−C−VIM Stroke 1.21 (1.03, 1.43) 12.00
Huang, et al. (2021) HDL−C−VIM Stroke 1.62 (0.94, 2.77) 1.85
Overall (I−squared = 91.6%, p = 0.000) 1.18 (1.09, 1.27) 100.00
NOTE: Weights are from random effects analysis
.7
1
2.5
HDL-C-Other
Han, et al. (2020) HDL−C−ARV MI 1.05 (1.02, 1.08) 38.78
Han, et al. (2020) HDL−C−ARV Stroke 1.08 (1.06, 1.11) 59.64
Liu, et al. (2020) HDL−C−ARV MI 1.17 (0.87, 1.57) 0.37
Wang, et al. (2020) HDL−C−ARV Stroke 1.08 (0.91, 1.28) 1.09
Huang, et al. (2021) HDL−C−ASV Stroke 1.33 (0.79, 2.24) 0.12
Overall (I−squared = 0.0%, p = 0.501) 1.07 (1.05, 1.09) 100.00
NOTE: Weights are from random effects analysis
.7
1
2.5

Figure S2. Forest plots of standardized RRs for different types and metrics of HDL-C variability (top vs. bottom quartile) and CVDs

Notes: CVDs, cardiovascular diseases; RR, relative risk; CI, confidence interval; HDL-C, high-density lipoprotein cholesterol; HDL-C-CV, coefficient of variation of HDL-C; HDL-C-SD, standard deviation of HDL-C; HDL-C-VIM, variation independent of mean of HDL-C; HDL-C-Other included average real variability of HDL-C (HDL-C-ARV); MI, myocardial infarction; AF, atrial fibrillation.

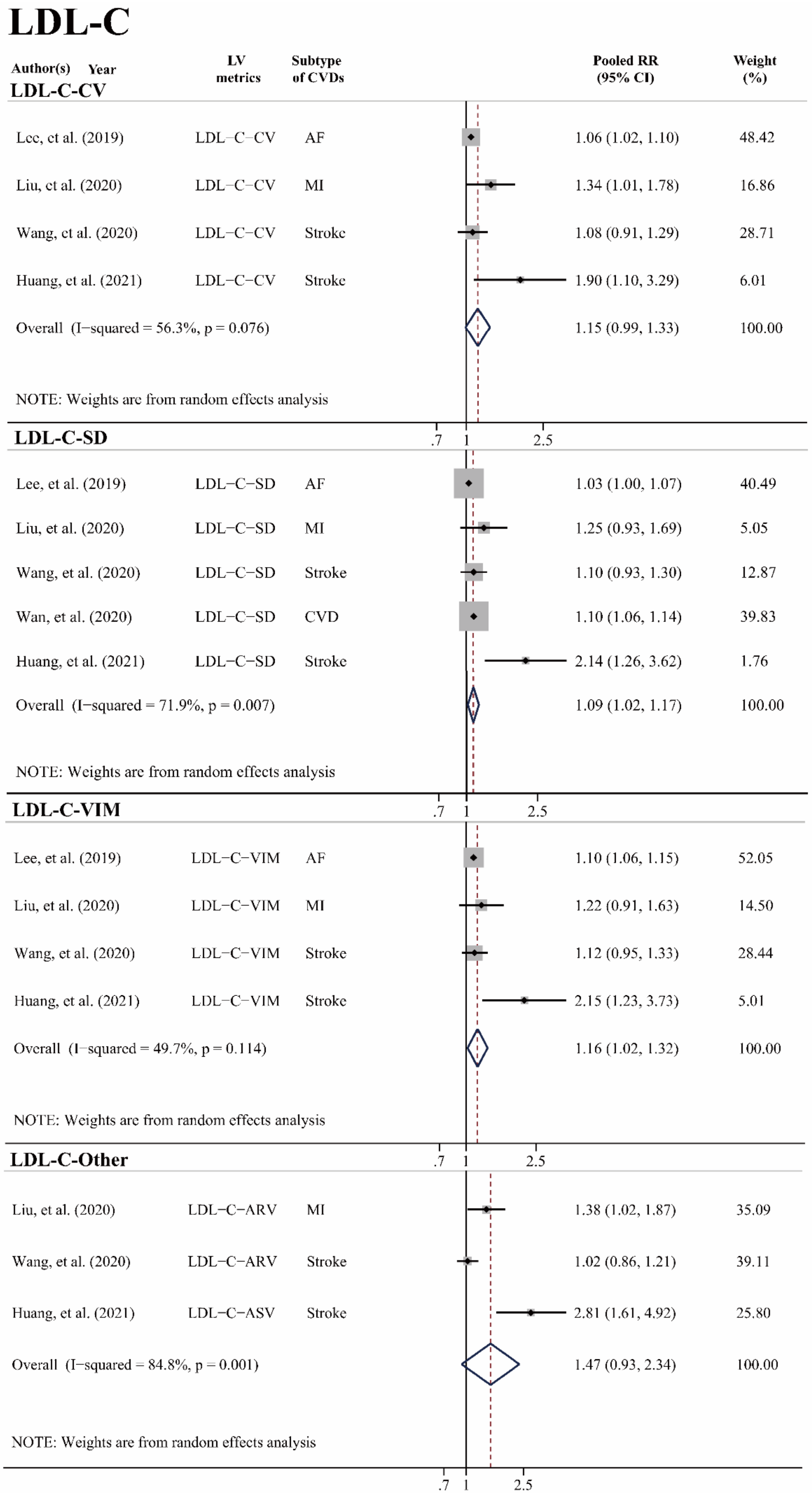
LDL-C
Author(s) Year
LV metrics
Subtype of CVDs
Pooled RR (95% CI)
Weight (%)
LDL-C-CV
Lee, et al. (2019) LDL−C−CV AF 1.06 (1.02, 1.10) 48.42
Liu, et al. (2020) LDL−C−CV MI 1.34 (1.01, 1.78) 16.86
Wang, et al. (2020) LDL−C−CV Stroke 1.08 (0.91, 1.29) 28.71
Huang, et al. (2021) LDL−C−CV Stroke 1.90 (1.10, 3.29) 6.01
Overall (I−squared = 56.3%, p = 0.076) 1.15 (0.99, 1.33) 100.00
NOTE: Weights are from random effects analysis
.7 1 2.5
LDL-C-SD
Lee, et al. (2019) LDL−C−SD AF 1.03 (1.00, 1.07) 40.49
Liu, et al. (2020) LDL−C−SD MI 1.25 (0.93, 1.69) 5.05
Wang, et al. (2020) LDL−C−SD Stroke 1.10 (0.93, 1.30) 12.87
Wan, et al. (2020) LDL−C−SD CVD 1.10 (1.06, 1.14) 39.83
Huang, et al. (2021) LDL−C−SD Stroke 2.14 (1.26, 3.62) 1.76
Overall (I−squared = 71.9%, p = 0.007) 1.09 (1.02, 1.17) 100.00
NOTE: Weights are from random effects analysis
.7 1 2.5
LDL-C-VIM
Lee, et al. (2019) LDL−C−VIM AF 1.10 (1.06, 1.15) 52.05
Liu, et al. (2020) LDL−C−VIM MI 1.22 (0.91, 1.63) 14.50
Wang, et al. (2020) LDL−C−VIM Stroke 1.12 (0.95, 1.33) 28.44
Huang, et al. (2021) LDL−C−VIM Stroke 2.15 (1.23, 3.73) 5.01
Overall (I−squared = 49.7%, p = 0.114) 1.16 (1.02, 1.32) 100.00
NOTE: Weights are from random effects analysis
.7 1 2.5
LDL-C-Other
Liu, et al. (2020) LDL−C−ARV MI 1.38 (1.02, 1.87) 35.09
Wang, et al. (2020) LDL−C−ARV Stroke 1.02 (0.86, 1.21) 39.11
Huang, et al. (2021) LDL−C−ASV Stroke 2.81 (1.61, 4.92) 25.80
Overall (I−squared = 84.8%, p = 0.001) 1.47 (0.93, 2.34) 100.00
NOTE: Weights are from random effects analysis
.7 1 2.5

Figure S3. Forest plots of standardized RRs for different types and metrics of LDL-C variability (top vs. bottom quartile) and CVDs

Notes: CVDs, cardiovascular diseases; RR, relative risk; CI, confidence interval; LDL-C, low-density lipoprotein cholesterol; LDL-C-CV, coefficient of variation of LDL-C; LDL-C-SD, standard deviation of LDL-C; LDL-C-VIM, variation independent of mean of LDL-C; LDL-C-Other included average real variability of LDL-C (LDL-C-ARV); MI, myocardial infarction; AF, atrial fibrillation.

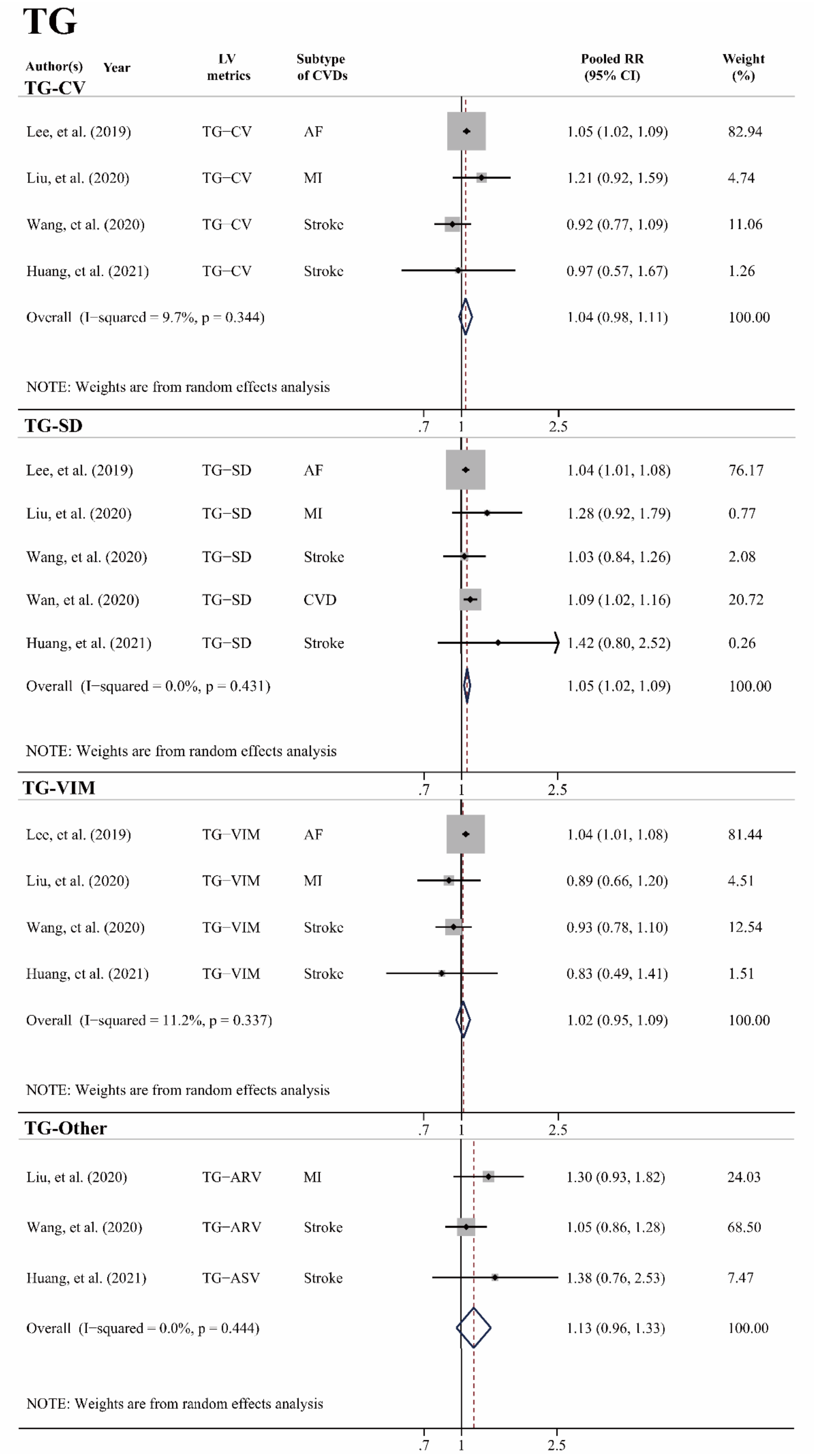
TG
Author(s) Year
LV metrics
Subtype of CVDs
Pooled RR (95% CI)
Weight (%)
TG-CV
Lee, et al. (2019) TG−CV AF 1.05 (1.02, 1.09) 82.94
Liu, et al. (2020) TG−CV MI 1.21 (0.92, 1.59) 4.74
Wang, et al. (2020) TG−CV Stroke 0.92 (0.77, 1.09) 11.06
Huang, et al. (2021) TG−CV Stroke 0.97 (0.57, 1.67) 1.26
Overall (I−squared = 9.7%, p = 0.344) 1.04 (0.98, 1.11) 100.00
NOTE: Weights are from random effects analysis
.7
1
2.5
TG-SD
Lee, et al. (2019) TG−SD AF 1.04 (1.01, 1.08) 76.17
Liu, et al. (2020) TG−SD MI 1.28 (0.92, 1.79) 0.77
Wang, et al. (2020) TG−SD Stroke 1.03 (0.84, 1.26) 2.08
Wan, et al. (2020) TG−SD CVD 1.09 (1.02, 1.16) 20.72
Huang, et al. (2021) TG−SD Stroke 1.42 (0.80, 2.52) 0.26
Overall (I−squared = 0.0%, p = 0.431) 1.05 (1.02, 1.09) 100.00
NOTE: Weights are from random effects analysis
.7
1
2.5
TG-VIM
Lee, et al. (2019) TG−VIM AF 1.04 (1.01, 1.08) 81.44
Liu, et al. (2020) TG−VIM MI 0.89 (0.66, 1.20) 4.51
Wang, et al. (2020) TG−VIM Stroke 0.93 (0.78, 1.10) 12.54
Huang, et al. (2021) TG−VIM Stroke 0.83 (0.49, 1.41) 1.51
Overall (I−squared = 11.2%, p = 0.337) 1.02 (0.95, 1.09) 100.00
NOTE: Weights are from random effects analysis
.7
1
2.5
TG-Other
Liu, et al. (2020) TG−ARV MI 1.30 (0.93, 1.82) 24.03
Wang, et al. (2020) TG−ARV Stroke 1.05 (0.86, 1.28) 68.50
Huang, et al. (2021) TG−ASV Stroke 1.38 (0.76, 2.53) 7.47
Overall (I−squared = 0.0%, p = 0.444) 1.13 (0.96, 1.33) 100.00
NOTE: Weights are from random effects analysis
.7
1
2.5

Figure S4. Forest plots of standardized RRs for different types and metrics of TG variability (top vs. bottom quartile) and CVDs

Notes: CVDs, cardiovascular diseases; RR, relative risk; CI, confidence interval; TG, triglycerides; TG-CV, coefficient of variation of TG; TG-SD, standard deviation of TG; TG-VIM, variation independent of mean of TG; TG-Other included average real variability of TG (TG-ARV); MI, myocardial infarction; AF, atrial fibrillation.

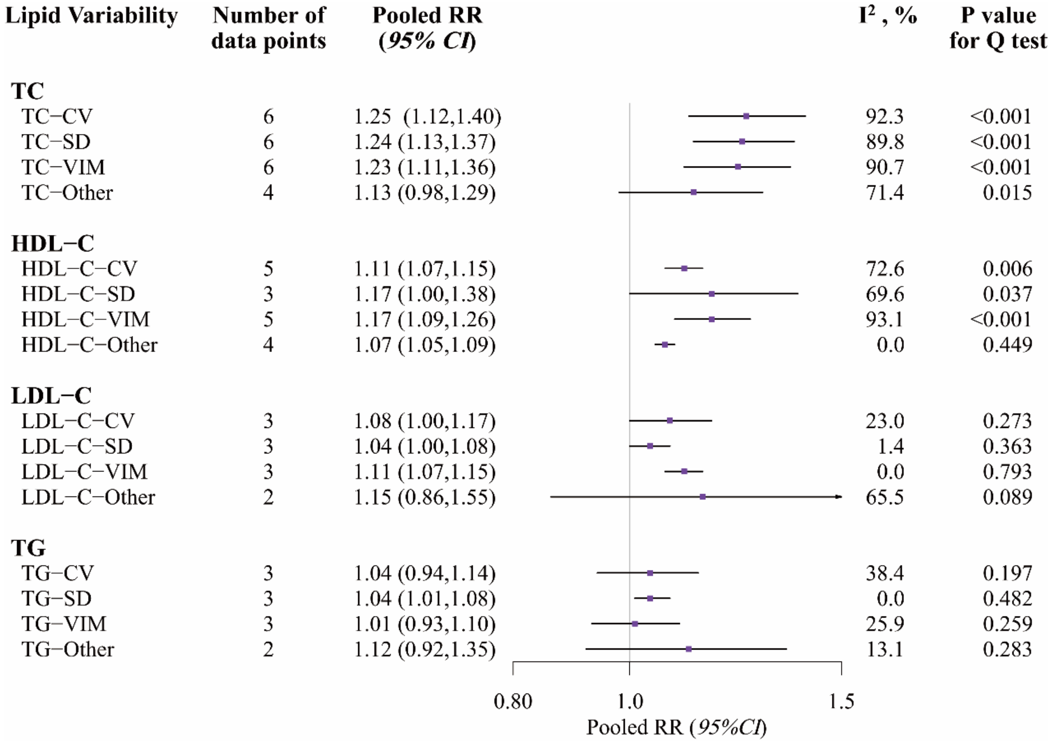

Figure S5. Sensitivity analysis of standardized RRs for different types and metrics of LV (top vs. bottom quartile) and CVDs with excluding the articles counted on patients with hypertension or diabetes

Notes: LV, lipid variability; CVDs, cardiovascular diseases; TC, total cholesterol; HDL-C, high-density lipoprotein cholesterol; LDL-C, low-density lipoprotein cholesterol; TG, triglycerides; CV, coefficient of variation; SD, standard deviation; VIM, variation independent of mean; TC-Other included average real variability of TC (TC-ARV), standard deviation of the residuals of TC (TC-SDR) and root mean square error of TC (TC-RMSE); HDL-C-Other included average real variability of HDL-C (HDL-C-ARV); LDL-C-Other included average real variability of LDL-C (LDL-C-ARV); TG-Other included average real variability of TG (TG-ARV).

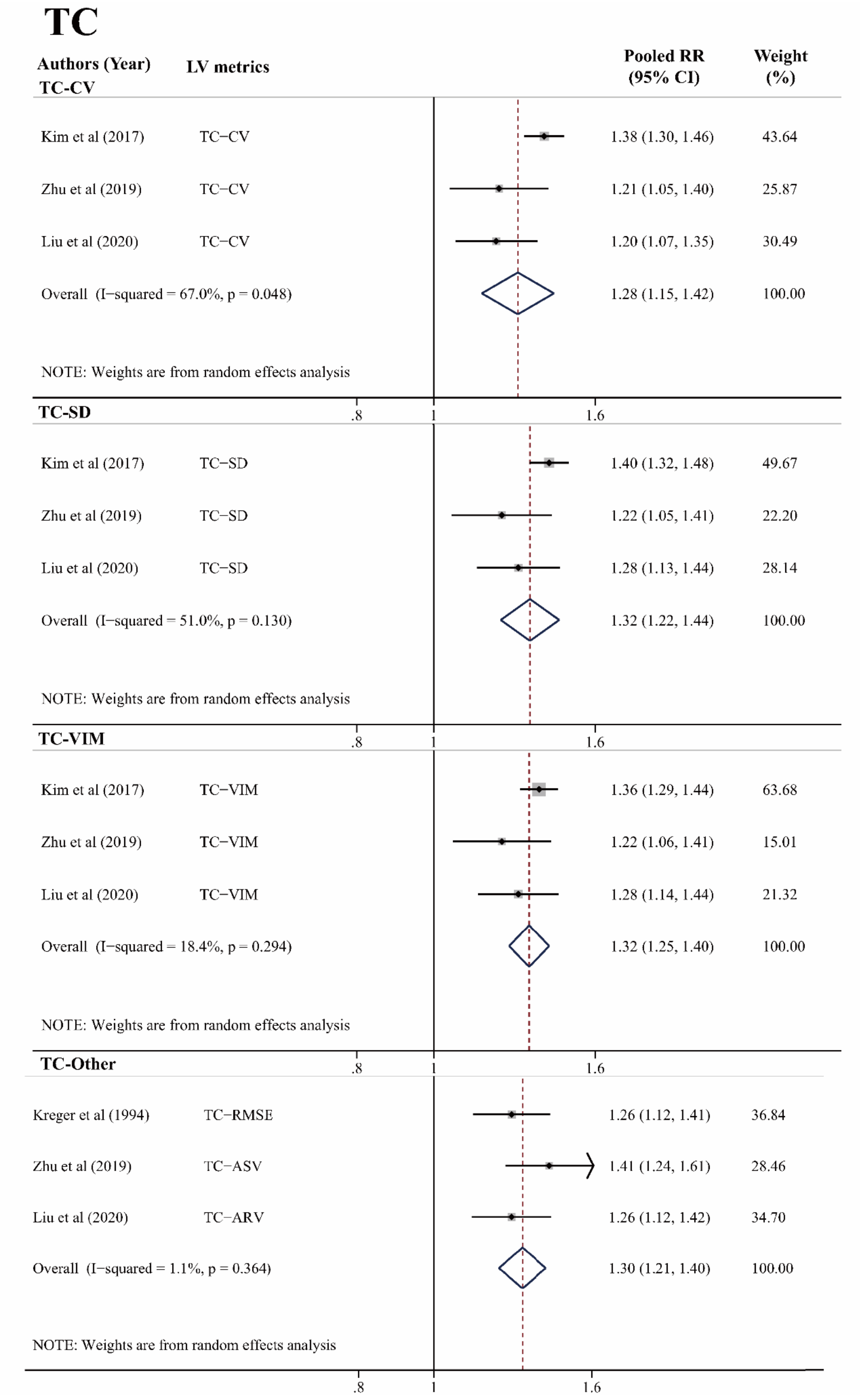
TC
Authors (Year)
LV metrics
Pooled RR (95% CI)
Weight (%)
TC-CV
Kim et al (2017) TC−CV 1.38 (1.30, 1.46) 43.64
Zhu et al (2019) TC−CV 1.21 (1.05, 1.40) 25.87
Liu et al (2020) TC−CV 1.20 (1.07, 1.35) 30.49
Overall (I−squared = 67.0%, p = 0.048) 1.28 (1.15, 1.42) 100.00
NOTE: Weights are from random effects analysis
.8
1
1.6
TC-SD
Kim et al (2017) TC−SD 1.40 (1.32, 1.48) 49.67
Zhu et al (2019) TC−SD 1.22 (1.05, 1.41) 22.20
Liu et al (2020) TC−SD 1.28 (1.13, 1.44) 28.14
Overall (I−squared = 51.0%, p = 0.130) 1.32 (1.22, 1.44) 100.00
NOTE: Weights are from random effects analysis
.8
1
1.6
TC-VIM
Kim et al (2017) TC−VIM 1.36 (1.29, 1.44) 63.68
Zhu et al (2019) TC−VIM 1.22 (1.06, 1.41) 15.01
Liu et al (2020) TC−VIM 1.28 (1.14, 1.44) 21.32
Overall (I−squared = 18.4%, p = 0.294) 1.32 (1.25, 1.40) 100.00
NOTE: Weights are from random effects analysis
.8
1
1.6
TC-Other
Kreger et al (1994) TC−RMSE 1.26 (1.12, 1.41) 36.84
Zhu et al (2019) TC−ASV 1.41 (1.24, 1.61) 28.46
Liu et al (2020) TC−ARV 1.26 (1.12, 1.42) 34.70
Overall (I−squared = 1.1%, p = 0.364) 1.30 (1.21, 1.40) 100.00
NOTE: Weights are from random effects analysis
.8
1
1.6

Figure S6. Forest plots of standardized RRs for different types and metrics of TC variability (top vs. bottom quartile) and all-cause mortality

Notes: LV, lipid variability; RR, relative risk; CI, confidence interval; TC, total cholesterol; TC-CV, coefficient of variation of TC; TC-SD, standard deviation of TC; TC-VIM, variation independent of mean of TC; TC-Other included average real variability of TC (TC-ARV), average successive variability of TC (TC-ASV) and root mean square error of TC (TC-RMSE).

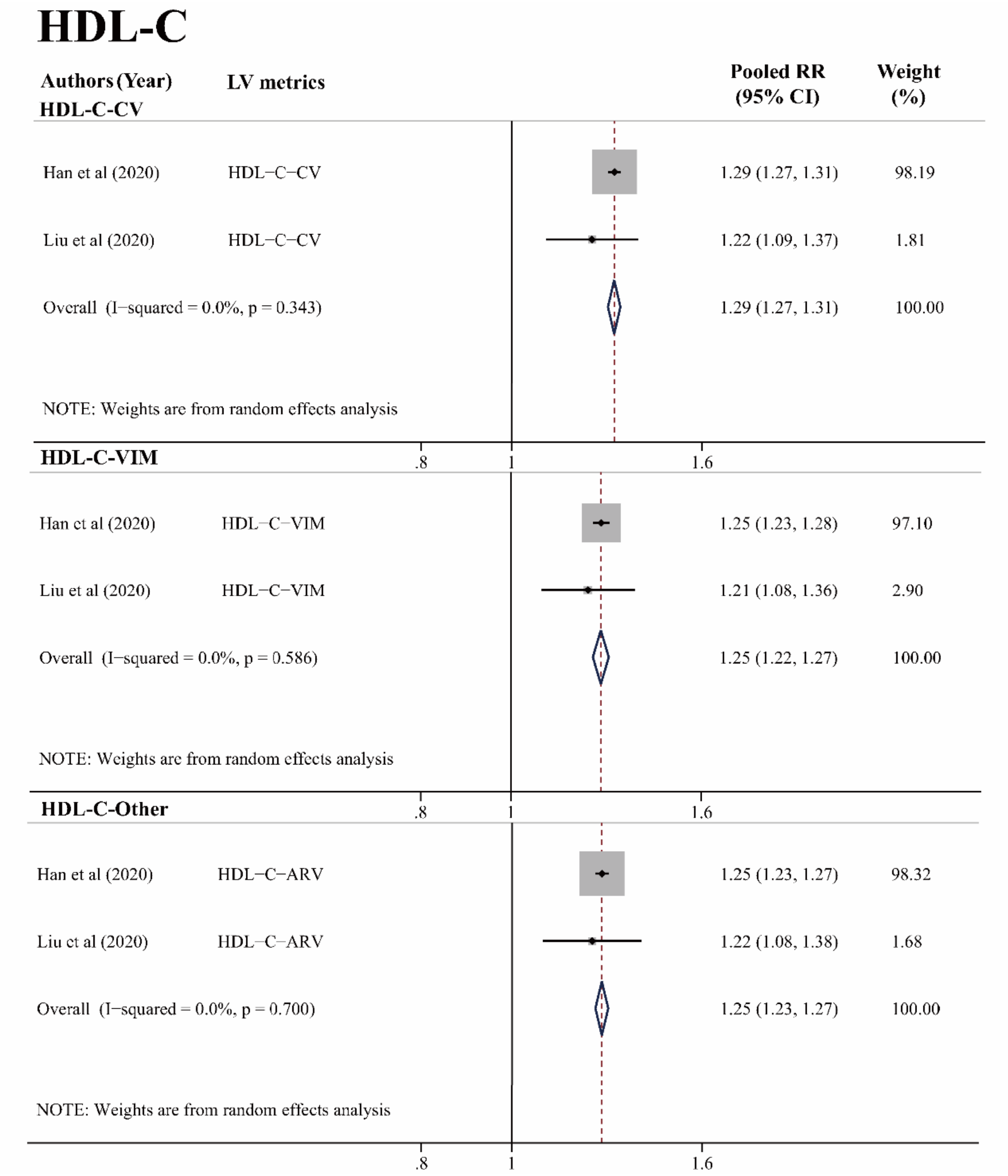

Figure S7. Forest plots of standardized RRs for different types and metrics of HDL-C variability (top vs. bottom quartile) and all-cause mortality

Notes: LV, lipid variability; RR, relative risk; CI, confidence interval; HDL-C, high-density lipoprotein cholesterol; HDL-C-CV, coefficient of variation of HDL-C; HDL-C-SD, standard deviation of HDL-C; HDL-C-VIM, variation independent of mean of HDL-C; HDL-C-Other included average real variability of HDL-C (HDL-C-ARV).

Lipid Variability	Number of data points	Pooled RR (*95% CI*)	I^2, %	P value for Q test
**TC**				
TC−CV	3	1.28 (1.15,1.42)	67.0	0.048
TC−SD	3	1.32 (1.22,1.44)	51.0	0.130
TC−VIM	3	1.32 (1.25,1.40)	18.4	0.294
TC−Other	3	1.30 (1.21,1.40)	1.1	0.364
**HDL−C**				
HDL−C−CV	2	1.29 (1.27,1.31)	0.0	0.343
HDL−C−SD	1	1.24 (1.09,1.41)	100.0	−
HDL−C−VIM	2	1.25 (1.22,1.27)	0.0	0.586
HDL−C−Other	2	1.25 (1.23,1.27)	0.0	0.700
**LDL−C**				
LDL−C−CV	1	1.19 (1.04,1.36)	100.0	−
LDL−C−SD	1	1.10 (0.97,1.24)	100.0	−
LDL−C−VIM	1	1.11 (0.98,1.26)	100.0	−
LDL−C−Other	1	1.05 (0.93,1.19)	100.0	−
**TG**				
TG−CV	1	1.05 (0.80,1.38)	100.0	−
TG−SD	1	1.03 (0.92,1.15)	100.0	−
TG−VIM	1	1.02 (0.90,1.15)	100.0	−
TG−Other	1	1.01 (0.87,1.18)	100.0	−

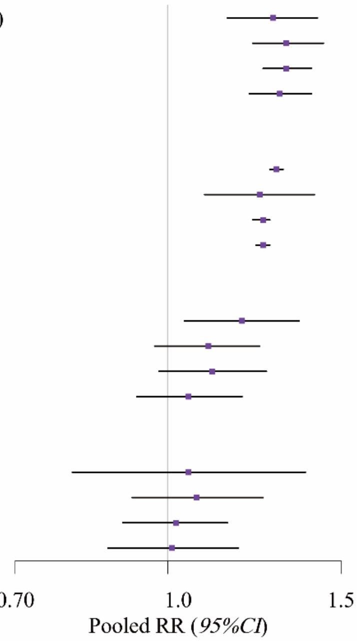

Figure S8. Sensitivity analysis of standardized RRs for different types and metrics of LV (top vs. bottom quartile) and all-cause mortality with excluding the articles counted on patients with hypertension or diabetes

Notes: LV, lipid variability; TC, total cholesterol; HDL-C, high-density lipoprotein cholesterol; LDL-C, low-density lipoprotein cholesterol; TG, triglycerides; CV, coefficient of variation; SD, standard deviation; VIM, variation independent of mean; TC-Other included average real variability of TC (TC-ARV), average successive variability of TC (TC-ASV) and root mean square error of TC (TC-RMSE); HDL-C-Other included average real variability of HDL-C (HDL-C-ARV); LDL-C-Other included average real variability of LDL-C (LDL-C-ARV); TG-Other included average real variability of TG (TG-ARV).

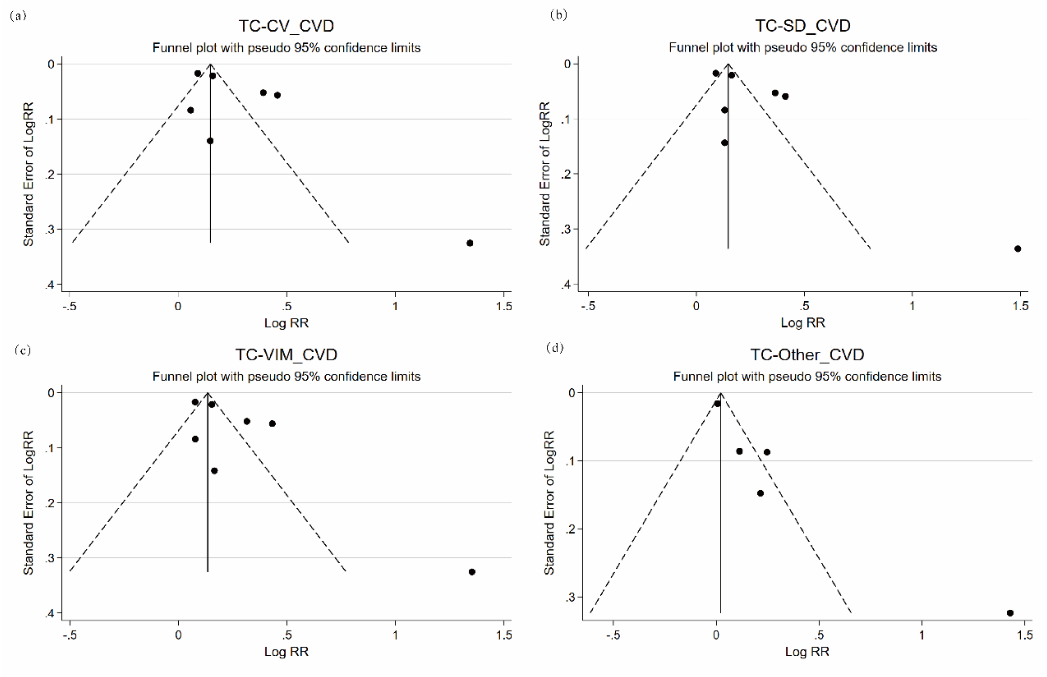

Figure S9. Funnel plots of standardized RRs for different types and metrics of TC variability (top vs. bottom quartile) and CVDs

Notes: CVDs, cardiovascular diseases; RR, relative risk; TC, total cholesterol; (a) TC-CV, coefficient of variation of TC; (b) TC-SD, standard deviation of TC; (c) TC-VIM, variation independent of mean of TC; (d) TC-Other included average real variability of TC (TC-ARV), standard deviation of the residuals of TC (TC-SDR) and root mean square error of TC (TC-RMSE).

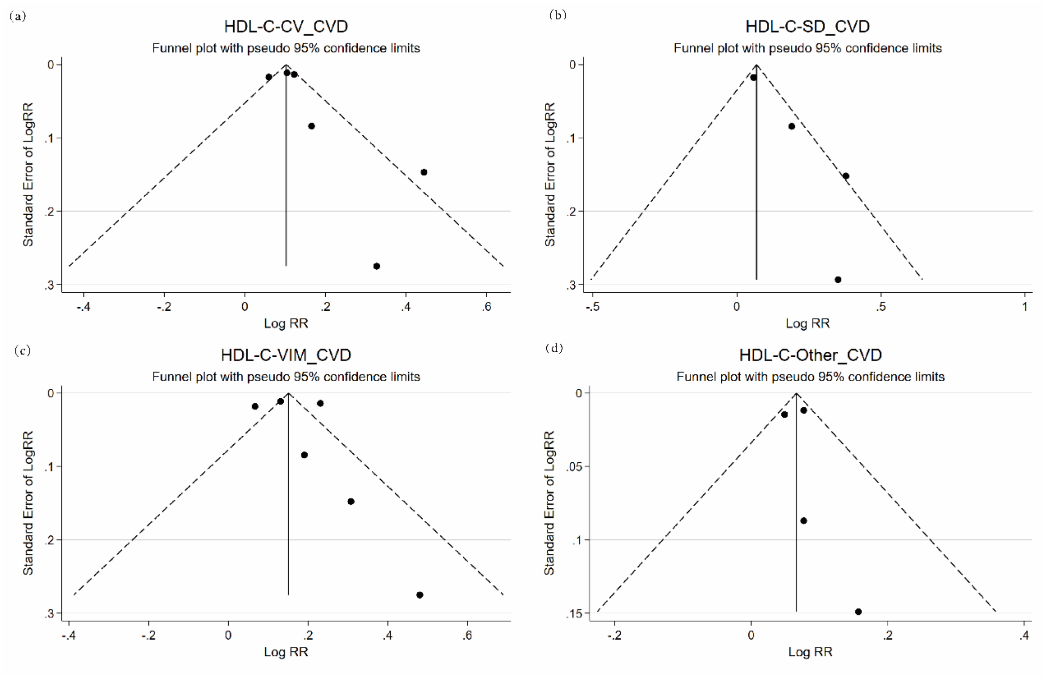

Figure S10. Funnel plots of standardized RRs for different types and metrics of HDL-C variability (top vs. bottom quartile) and CVDs

Notes: CVDs, cardiovascular diseases; RR, relative risk; HDL-C, high-density lipoprotein cholesterol; (a) HDL-C-CV, coefficient of variation of HDL-C; (b) HDL-C-SD, standard deviation of HDL-C; (c) HDL-C-VIM, variation independent of mean of HDL-C; (d) HDL-C-Other included average real variability of HDL-C (HDL-C-ARV).

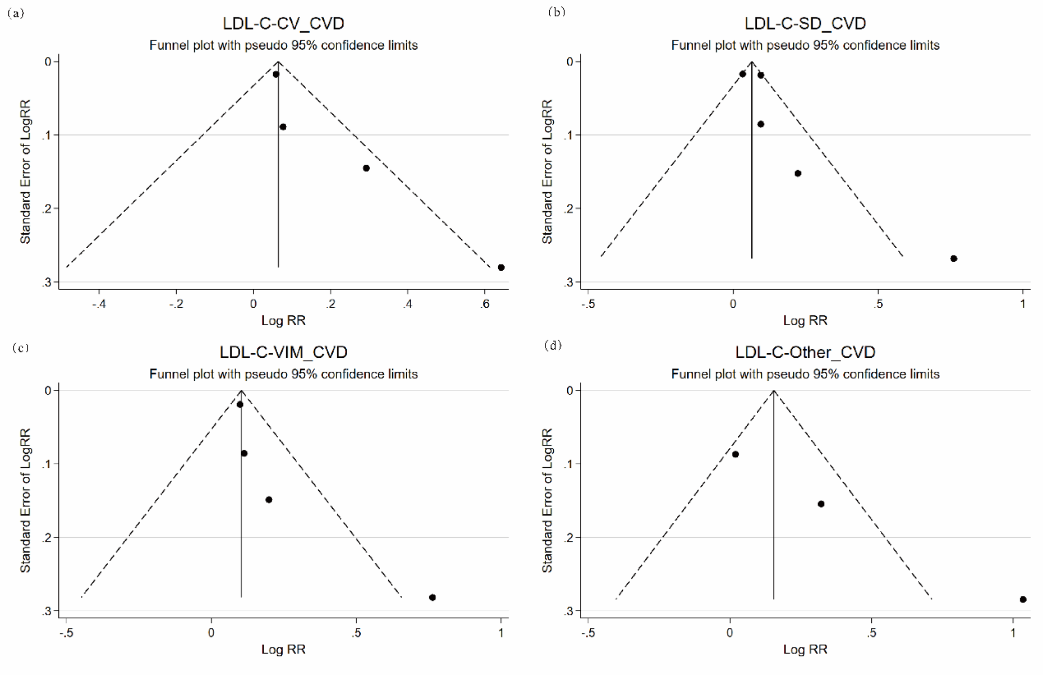

Figure S11. Funnel plots of standardized RRs for different types and metrics of LDL-C variability (top vs. bottom quartile) and CVDs

Notes: CVDs, cardiovascular diseases; RR, relative risk; LDL-C, low-density lipoprotein cholesterol; (a) LDL-C-CV, coefficient of variation of LDL-C; (b) LDL-C-SD, standard deviation of LDL-C; (c) LDL-C-VIM, variation independent of mean of LDL-C; (d) LDL-C-Other included average real variability of LDL-C (LDL-C-ARV).

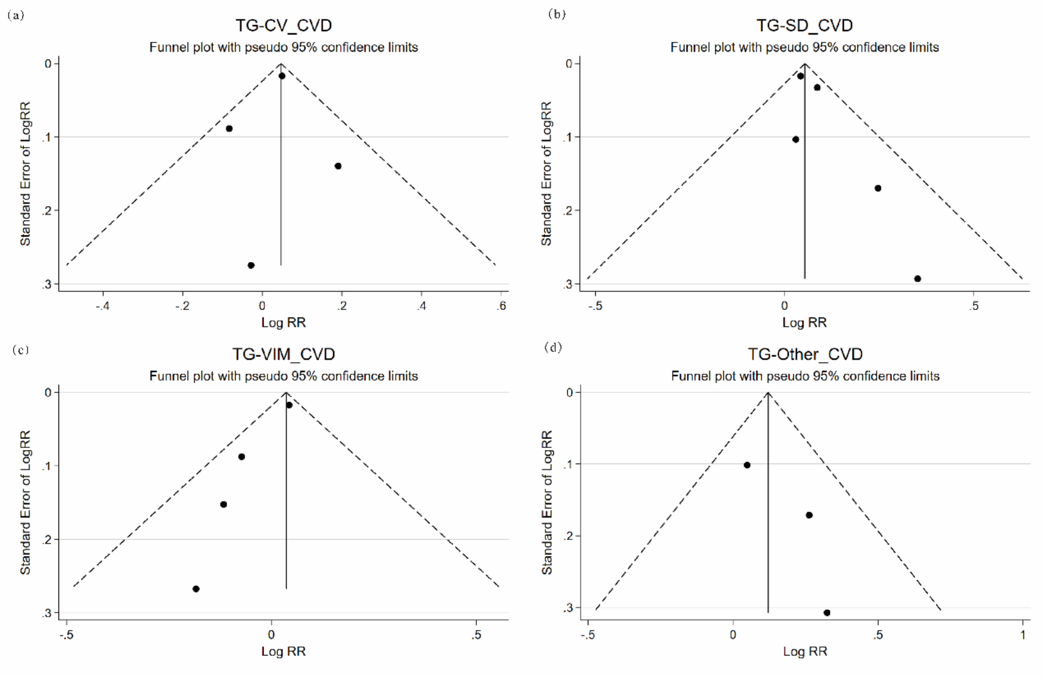

Figure S12. Funnel plots of standardized RRs for different types and metrics of TG variability (top vs. bottom quartile) and CVDs

Notes: CVDs, cardiovascular diseases; RR, relative risk; TG, triglycerides; (a) TG-CV, coefficient of variation of TG; (b) TG-SD, standard deviation of TG; (c) TG-VIM, variation independent of mean of TG; (d) TG-Other included average real variability of TG (TG-ARV).

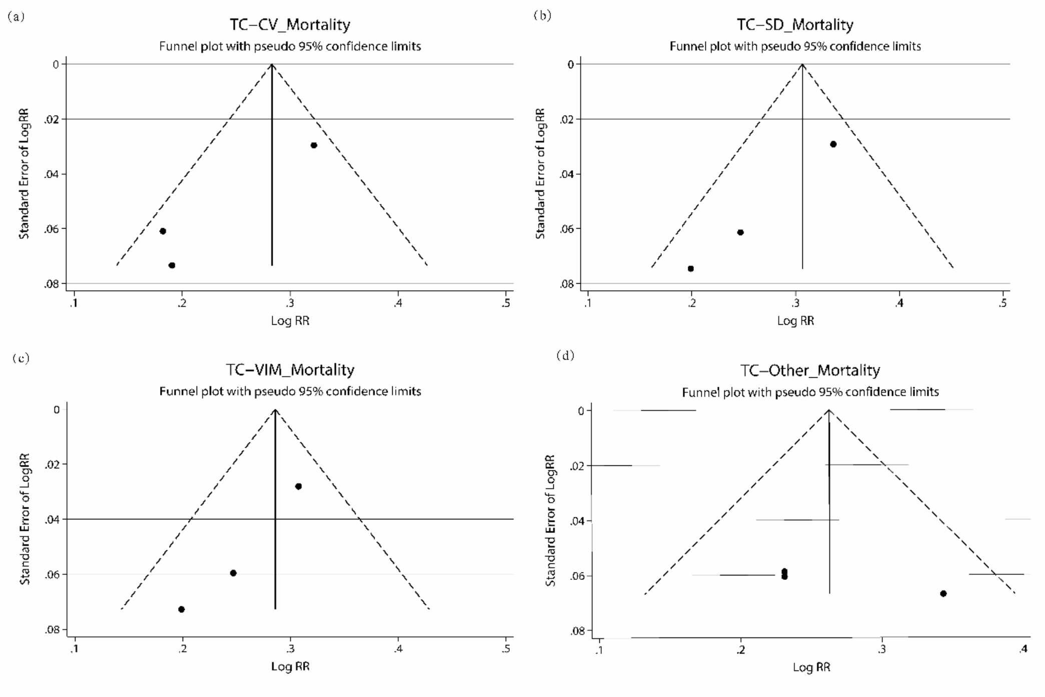

Figure S13. Funnel plots of standardized RRs for different types and metrics of TC variability (top vs. bottom quartile) and all-cause mortality

Notes: CVDs, cardiovascular diseases; RR, relative risk; TC, total cholesterol; (a) TC-CV, coefficient of variation of TC; (b) TC-SD, standard deviation of TC; (c) TC-VIM, variation independent of mean of TC; (d) TC-Other included average real variability of TC (TC-ARV), average successive variability of TC (TC-ASV) and root mean square error of TC (TC-RMSE).

Reference

1. Kreger, B.E.; Odell, P.M.; D'Agostino, R.B.; Wilson, P.F. Long-term intraindividual cholesterol variability: natural course and adverse impact on morbidity and mortality--the Framingham Study. *American heart journal* **1994**, *127*, 1607-1614.
2. Kim, M.K.; Han, K.; Kim, H.S.; Park, Y.M.; Kwon, H.S.; Yoon, K.H.; Lee, S.H. Cholesterol variability and the risk of mortality, myocardial infarction, and stroke: a nationwide population-based study. *European heart journal* **2017**, *38*, 3560-3566.
3. Kwon, S.; Lee, S.R.; Choi, E.K.; Lee, S.H.; Han, K.D.; Lee, S.Y.; Yang, S.; Park, J.; Choi, Y.J.; Lee, H.J., et al. Visit-to-visit variability of metabolic parameters and risk of heart failure: A nationwide population-based study. *International journal of cardiology* **2019**.
4. Zhu, Y.; Lu, J.M.; Yu, Z.B.; Li, D.; Wu, M.Y.; Shen, P.; Lin, H.B.; Wang, J.B.; Chen, K. Intra-individual variability of total cholesterol is associated with cardiovascular disease mortality: A cohort study. *Nutrition, metabolism, and cardiovascular diseases : NMCD* **2019**, *29*, 1205-1213.
5. Lee, H.J.; Lee, S.R.; Choi, E.K.; Han, K.D.; Oh, S. Low Lipid Levels and High Variability are Associated With the Risk of New-Onset Atrial Fibrillation. *Journal of the American Heart Association* **2019**, *8*, e01277.
6. Liu, X.; Wu, S.; Song, Q.; Wang, X. Visit-to-visit variability of lipid measurements and the risk of myocardial infarction and all-cause mortality: A prospective cohort study. *Atherosclerosis* **2020**, *312*, 110-116.
7. Han, B.H.; Han, K.; Yoon, K.H.; Kim, M.K.; Lee, S.H. Impact of Mean and Variability of High-Density Lipoprotein-Cholesterol on the Risk of Myocardial Infarction, Stroke, and Mortality in the General Population. *Journal of the American Heart Association* **2020**, *9*, e015493.
8. Kalani, R.; Bartz, T.M.; Suchy-Dicey, A.; Elkind, M.S.V.; Psaty, B.M.; Leung, L.Y.; Rice, K.; Tirschwell, D.; Longstreth, W.T., Jr. Cholesterol Variability and Cranial Magnetic Resonance Imaging Findings in Older Adults: The Cardiovascular Health Study. *Stroke* **2020**, *51*, 69-74.
9. Wang, A.; Li, H.; Yuan, J.; Zuo, Y.; Zhang, Y.; Chen, S.; Wu, S.; Wang, Y. Visit-to-Visit Variability of Lipids Measurements and the Risk of Stroke and Stroke Types: A Prospective Cohort Study. *Journal of stroke* **2020**, *22*, 119-129.
10. Wan, E.Y.F.; Yu, E.Y.T.; Chin, W.Y.; Barrett, J.K.; Mok, A.H.Y.; Lau, C.S.T.; Wang, Y.; Wong, I.C.K.; Chan, E.W.Y.; Lam, C.L.K. Greater variability in lipid measurements associated with cardiovascular disease and mortality: A 10-year diabetes cohort study. *Diabetes, obesity & metabolism* **2020**, *22*, 1777-1788.
11. Huang, Y.Q.; Liu, L.; Liu, X.C.; Lo, K.; Tang, S.T.; Feng, Y.Q.; Zhang, B. The association of blood lipid parameters variability with ischemic stroke in hypertensive patients. *Nutrition, metabolism, and cardiovascular diseases : NMCD* **2021**, *31*, 1521-1532.
